# Acrophobia and visual height intolerance: advances in epidemiology and mechanisms

**DOI:** 10.1007/s00415-020-09805-4

**Published:** 2020-05-22

**Authors:** Doreen Huppert, Max Wuehr, Thomas Brandt

**Affiliations:** 1 German Center for Vertigo and Balance Disorders and Institute for Clinical Neurosciences, Ludwig Maximilians Universität, Marchioninistr. 15, 81377 Munich, Germany; 2 German Center for Vertigo and Balance Disorders, Ludwig Maximilians Universität, Marchioninistr. 15, 81377 Munich, Germany; 3 Department of Neurology, Universitätklinik München Ludwig Maximilians Universität, Marchioninistr. 15, 81377 Munich, Germany

**Keywords:** Acrophobia, Fear of heights, Visual height intolerance, Balance control, Muscle co-contraction

## Abstract

Historical descriptions of fear at heights date back to Chinese and Roman antiquity. Current definitions distinguish between three different states of responses to height exposure: a physiological height imbalance that results from an impaired visual control of balance, a more or less distressing visual height intolerance, and acrophobia at the severest end of the spectrum. Epidemiological studies revealed a lifetime prevalence of visual height intolerance including acrophobia in 28% of adults (32% in women; 25% in men) and 34% among prepubertal children aged 8–10 years without gender preponderance. Visual height intolerance first occurring in adulthood usually persists throughout life, whereas an early manifestation in childhood usually shows a benign course with spontaneous relief within years. A high comorbidity was found with psychiatric disorders (e.g. anxiety and depressive syndromes) and other vertigo syndromes (e.g. vestibular migraine, Menière’s disease), but not with bilateral vestibulopathy. Neurophysiological analyses of stance, gait, and eye movements revealed an anxious control of postural stability, which entails a co-contraction of anti-gravity muscles that causes a general stiffening of the whole body including the oculomotor apparatus. Visual exploration is preferably reduced to fixation of the horizon. Gait alterations are characterized by a cautious slow walking mode with reduced stride length and increased double support phases. Anxiety is the critical factor in visual height intolerance and acrophobia leading to a motor behavior that resembles an atavistic primitive reflex of feigning death. The magnitude of anxiety and neurophysiological parameters of musculoskeletal stiffening increase with increasing height. They saturate, however, at about 20 m of absolute height above ground for postural symptoms and about 40 m for anxiety (70 m in acrophobic participants). With respect to management, a differentiation should be made between behavioral recommendations for prevention and therapy of the condition. Recommendations for coping strategies target behavioral advices on visual exploration, control of posture and locomotion as well as the role of cognition. Treatment of severely afflicted persons with distressing avoidance behavior mainly relies on behavioral therapy.

## Introduction

About one-third of the general population suffers from susceptibility to acrophobia and visual height intolerance, a distressing condition that reduces quality of life, and causes behavioral constraints and phobic avoidance of exposure to heights. Epidemiological surveys and experimental studies revealed new insights into risk factors, triggers, signs, and symptoms as well as the underlying mechanisms, which allow effective recommendations for behavioral coping strategies and behavioral therapy using real and virtual height stimuli to be made. Irrational anxiety plays a major role in the condition of acrophobia and visual height intolerance, which Balaban and Jacob [1] stated in their seminal historical article “Background and history of the interface between anxiety and vertigo”. This vertigo-balance-anxiety interface has been an integral component of medical literature since antiquity.

## Historical descriptions of vertigo and fear at heights in Chinese and Roman antiquity

**Fig. 1:**
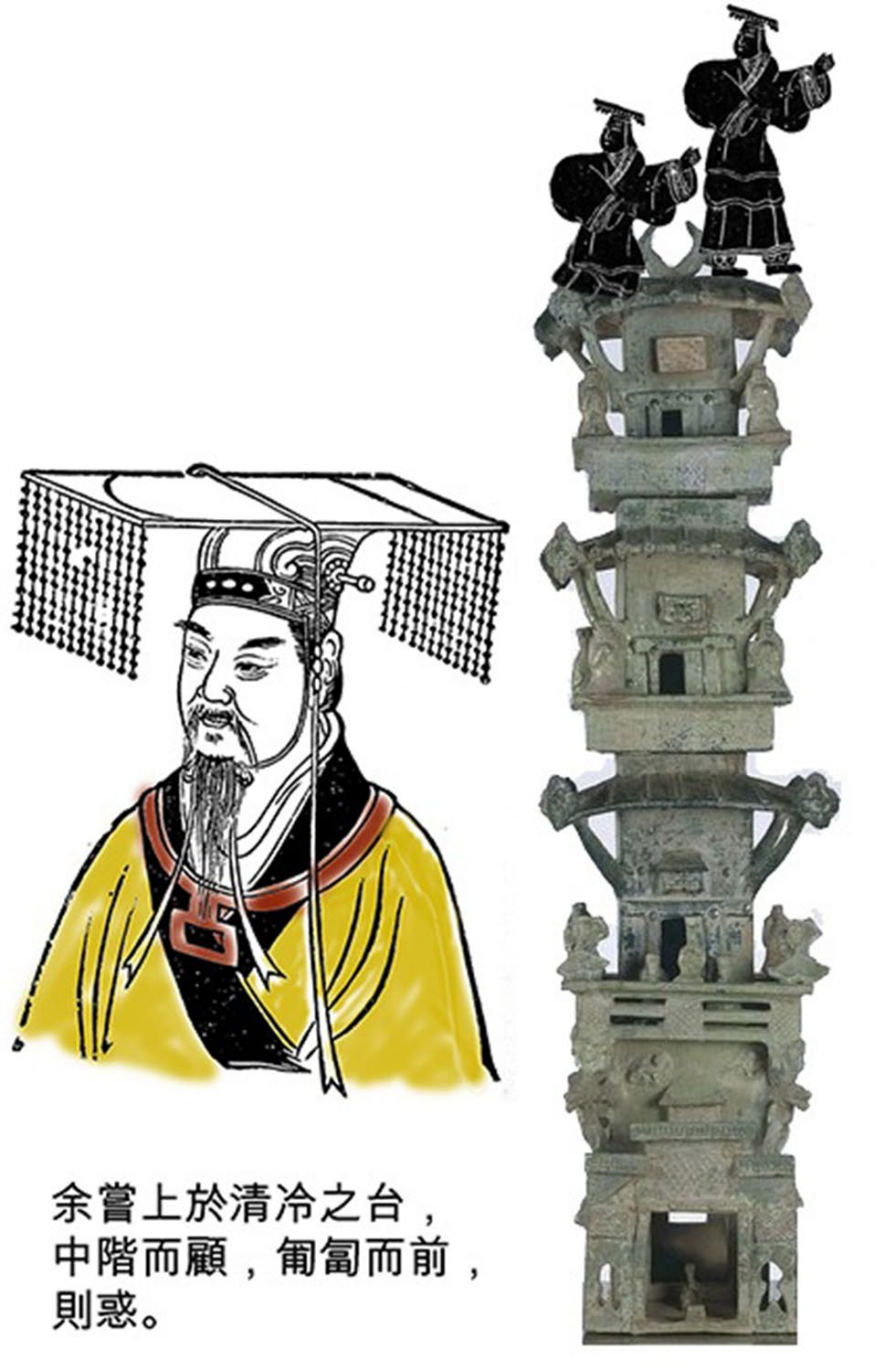
Picture of the Yellow Thearch (“the yellow emperor”) with a citation from the book Huangdi Neijing [67]. On the right, a typical Chinese watchtower from Han Dynasty, Henan Province, first—second century AD (Art Gallery of New South Wales). The emperor suffered from symptoms that correspond to the modern concept of the syndrome of fear of heights when he climbed onto a clear, cold observation platform. He found out that kneeling down reduces these symptoms, a behavior which was later confirmed in psychophysical experiments: symptoms of fear of heights were strongest during free upright stance, moderate when the subject kneeled, and absent when the subject lied when looking down [3]

In chapter 80 of the book Huangdi Neijing Lingshu—written between the second century BC and the second century AD—part of ‘The internal classic of the Yellow Thearch’—the emperor Huang Di described how he often felt uncomfortable and confused when climbing a watchtower observation platform. In Chinese medicine of correspondences, the critical stimulus was not considered ‘visual height’, but the life essence Qi, which when cooled with increasing height triggered a dizziness of the eyes as well as winds that were able to penetrate the neck [2]. The emperor found out that kneeling down reduces symptoms (Fig. 1), an observation which was rediscovered later in psychophysical experiments [3]. Sources for fear at heights can also be found in antique Roman texts. In his work 'Ab urbe condita', Livius (59 BC–17 AD) described that soldiers dropped down from high ladders when trying to surmount city walls which impeded the conquest of Carthago Nova. A second example can be found in Ovid’s (43 BC–17 AD) ‘Metamorphoses’, in which the characteristic behavior of the partridges, which avoid the height, is explained mythologically by an accident in the world of the antique gods when the goddess Athena caught Perdix (the Latin word for partridge) who was hurled by his uncle from the Acropolis and metamorphosed him into a bird of the same name, the partridge [4]. More recently, in the eighteenth century, Erasmus Darwin, the grandfather of Charles Darwin, was the first to hypothesize that sensorimotor mechanisms cause postural and gait imbalance when exposed to heights [5, 6].

## Definitions and grading of susceptibility

The term ‘fear of heights’ implies an anticipatory fear that leads to the avoidance of heights and thus prevents anxiety attacks and catastrophic falls. Psychiatrists use the term to define acrophobia, a specific phobia according to the classification schemes of ICD-10 (International Classification of Diseases) and DSM-V (Diagnostic and Statistical Manual of Mental Disorders) which requires psychotherapy [7, 8]. The nonmedical Anglo-American community uses the same term to refer to a less pronounced visual height intolerance that does not fulfill the criteria of a specific phobia.

To overcome this conceptual confusion and to clearly distinguish between physiological and psychopathological mechanisms at heights, the following three terms have been proposed to define the different states of responses to height exposure (Table 1) [9, 10]:

1. A physiological height imbalance of posture that results from an impaired visual control of stance and gait when the distance to stationary surroundings becomes too large for visual detection and counteraction of body movements [3];
2. A more or less distressing, stimulus-dependent visual height intolerance, which causes the apprehension of losing balance or falling, but does not meet the criteria of a specific phobia.
3. Fear of heights or acrophobia, a specific phobia representing the severest end of the spectrum.

**Table 1:** Forms of physiological and psychopathological conditions active during exposure to visual heights (modified from [10])

Term	Prevalence	Mechanism	Clinical relevance
Physiological visual height imbalance	100%	Impaired visual control of postural balance	None
Visual height intolerance	28%	Distress and anxiety when exposed to heights	50% of those afflicted
Acrophobia (fear of heights)	3–6%	Specific phobia	100% (requires psychotherapy)

Common questionnaires to validate susceptibility to fear of heights either compare self-reports and overt-behavioral procedures [11] or measure height-relevant interpretation biases to assess the relationship between biased interpretations and symptoms of acrophobia [12]. On the basis of various epidemiological and phenomenological studies, a short questionnaire was developed and validated that allows (1) a continuous quantification of the severity of visual height intolerance within a metric interval scale from 0 to 13 and (2) differentiation of the diagnosis of acrophobia by including two additional questions [13].

## Epidemiology and susceptibility across life span

Most available epidemiological studies focus on symptoms of fear of heights (acrophobia) mainly based on features of panic attacks. The frequency of acrophobia has a lifetime prevalence of 3.1–6.4% [14–21]. In two representative Germany-wide studies (*n* = 3517, *n* = 2012), the lifetime prevalence of the more broadly defined visual height intolerance was 28% in adults, slightly higher in women with 32% than in men (25%). The risk of developing visual height intolerance is higher in those individuals with a positive family history of vertigo, with additional illnesses such as motion sickness, Menière’s disease (9%), anxiety disorders, or migraine (21%) [22, 23]. The majority of susceptible individuals experience a chronic unfavorable course of illness, which is particularly associated with the presence of coexisting major depression, chronic fatigue, panic attacks, initial traumatic trigger, social phobia, other specific phobic fears, and female sex [23]. Visual height intolerance can develop throughout the lifespan, but most often (30%) occurs for the first time during the second decade of life. The main symptoms during height exposure in predisposed persons include anxiety, to-and-fro vertigo, unsteadiness and gait insecurity, weak knees, inner agitation and vegetative symptoms such as rapid heartbeat, sweating, drowsiness and tremor. The most common trigger situations are looking down from towers, followed by hiking and mountaineering, climbing ladders, walking over a bridge and looking down from a high-rise window. For about half of affected individuals, the trigger situations generalize in the further course of susceptibility (57%). A coping strategy for over half of those affected is to avoid triggering situations, which leads to restrictions in daily activities and reduced quality of life [22, 24].

Psychiatric evaluations revealed that in 22.5% of susceptible individuals, symptoms may from time to time worsen to the intensity of panic attacks. There is high comorbidity with anxiety disorders (16.7%) and depressive syndromes (26.1%), but not with other somatoform disorders [23]. The same psychiatric comorbidity has been reported for almost half of patients with other vertigo/dizziness syndromes, for example vestibular migraine or Menière’s disease [25]. In another study, the prevalence of susceptibility to visual height intolerance and fear of heights was found to be increased in various groups of vestibular diseases, including phobic postural vertigo/functional dizziness (64%), vestibular migraine (61%,), vestibular paroxysmia (56%), benign paroxysmal positioning vertigo (54%), unilateral vestibulopathy (49%), and Menière’s disease (48%), but was normal compared to the general population in patients with bilateral vestibulopathy (29%) [26]. A possible explanation for the latter observation is that patients with bilateral vestibulopathy may expose themselves less frequently to heights because of their inherent postural instability [26]. Associations are known to exist between anxiety disorders and alcohol-drinking behavior [27–30]. This kind of comorbidity was also shown for specific phobias in a large representative epidemiological survey in the United States [20] with varying frequencies among subtypes of specific phobias [18, 31]: it was higher for animal, situational, and blood/injury subtypes as compared to environmental subtypes [15, 17]. In contrast, there is no evidence that visual height intolerance and fear of heights are significantly associated with alcohol misuse [32]. However, up to 30% of individuals with fear of heights report the use of anxiolytic medication or the consumption of alcohol for the prevention of or relief from anxiety at heights [33–35]. With respect to self-efficacy, a cross-sectional survey revealed a lower level of general self-confidence in susceptible individuals, in particular in those who do not actively seek help or expose themselves to height situations [36].

## Special features of visual height intolerance in children

Epidemiological studies on children are available for anxiety disorders and specific phobias, especially fear of the dark, spider phobia, and medical phobias as well as for fear of heights, however, without detailed specification of its prevalence [37–39]. A survey among prepubertal children aged 8–10 years on the frequency and phenomenology of visual height intolerance and fear of heights revealed a prevalence of 34% with no gender preponderance [40]. The mean age at onset of symptoms was 5.9 years. Triggers and symptoms in children were similar to those of adults. Avoidance behavior was reported by less than one-third of the children without major impairment of quality of life. An essential difference of visual height intolerance in childhood is the good prognosis with spontaneous remission in up to 48% until the age of 10. Since corresponding epidemiological studies in adults found only 4.5% of individuals who reported an emergence of symptoms when exposed to heights during their first decade of life [22], it can be hypothesized that two separate courses of the development of susceptibility may exist: an early beginning visual height intolerance that usually resolves spontaneously in contrast to a, in most cases, persistent form that occurs during adulthood [23, 40]. Fear-related to height stimuli can be assumed to be a protective mechanism that restrains children from potentially harmful situations, with which they are yet too immature to cope. However, active hazard of height exposure during maturation may have an anti-phobic effect [41]. One could argue that a controlled exposure to the feared or avoided situations represents a successful strategy of desensitization, whereas a too restrictive behavior may promote persistence of the condition [40].

## Mechanisms

Reported mental and physical symptoms of height intolerant individuals are instability of gait and stance, heavy or stunned legs, and vertigo. Three typical reports from transcribed interviews are [24]:

“Well, I really think at that point, I’m a goner right now. So like I settle my affairs then and there, because I think, I won’t survive this.”

“…and when going down across a scree slope, which was quite steep, I simply couldn’t go any further. It’s like a mental block – I have to sit down, I’m overwhelmed by fear, my heartbeat begins racing so fast that I also feel like I’m suffocating, I can’t breathe anymore…”

“Yeah, and when it gets really bad, then I can't even lift my foot. It’s like my feet are glued to the ground…”

## Experimental studies of gait, stance, and gaze behavior under real height stimulation

Increased postural threat, for instance while walking on a modestly elevated support surface in the laboratory, causes gait changes especially in older people. These changes are characterized by a reduced speed of walking, shorter steps, decreased cadence, and longer times in double support [42–44]. Analogously, standing on elevated surfaces in the laboratory alters static postural control in particular a musculoskeletal stiffening of the postural control apparatus [45]. These postural alterations are accompanied by changes in vestibulospinal balance reflexes in terms of a greater coupling between vestibular inputs and body sway responses [46]. This threat-induced modulation of vestibulospinal reflexes is presumably closely related to postural stiffening by anti-gravity muscle co-contraction. An analogous modulation of sensorimotor balance reflexes can be observed while standing on the ground when balance is threatened by unpredictable tilts of the support surface [47]. This finding suggests, that alterations in sensorimotor balance control at heights are primarily elicited by fear and anxiety rather than by the visual height stimulus alone. Correspondingly, personality traits measured by questionnaires on anxiety and willingness to take physical risks are associated with postural alterations when standing at an elevated platform edge [48]. Moreover, acrophobic individuals have a poorer postural performance in static and dynamic balance tasks [49].

**Fig. 2:**
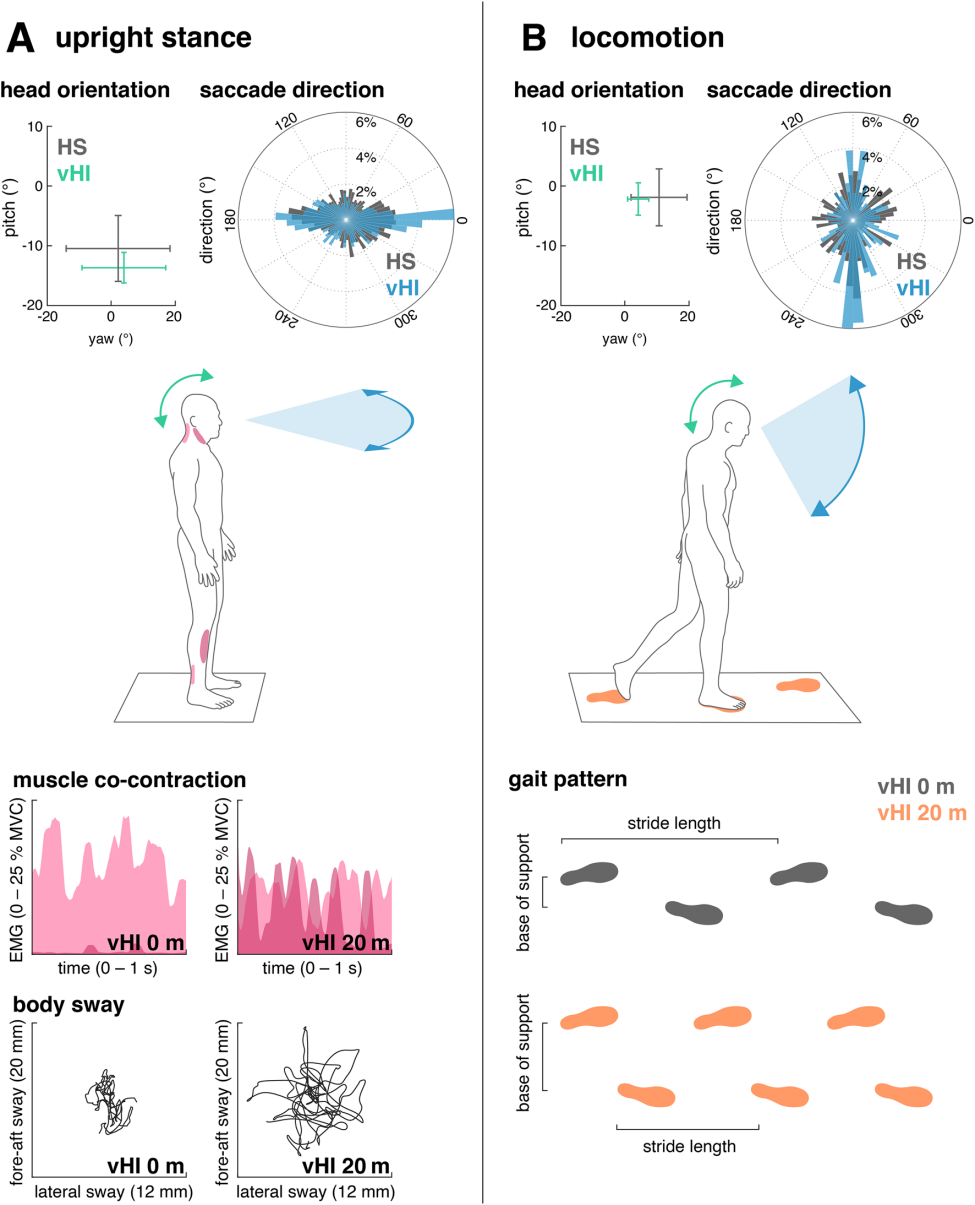
Overview of height-induced alterations of visual exploration, postural and locomotion control in individuals with visual height intolerance (vHI) and healthy insusceptible subjects (HS) while being exposed to heights on an emergency balcony 20 m above ground. **a** Behavioral alterations during quiet upright stance. **b** Behavioral alterations during locomotion. Top panel: group means and interquartile ranges of head orientation and histograms of the direction of corresponding saccadic eye movements. During both standing and walking, individuals with vHI show considerably reduced head movements. Saccadic eye movements during stance in individuals with vHI are preferably directed along the horizontal plane. In contrast, during locomotion, they perform saccades primarily along the vertical plane. Bottom panel: during height exposure, postural control in individuals with vHI is characterized by increased co-contraction of anti-gravity muscles and increased body sway amplitudes. Locomotion is characterized by a slow and cautious mode of walking, with a reduced stride length and a broadened base of support

The above-described studies were performed preferably in the laboratory and tested balance function under threat conditions in healthy subjects. Neurophysiological studies on sensorimotor control in individuals with more or less severe visual height intolerance including acrophobia have been conducted during real exposure to heights (Fig. 2). These experiments were performed on an open escape balcony at a height of 20 m above ground level with a focus on either visual exploration behavior by use of mobile infrared eye-tracking goggles with integrated inertial sensors for monitoring of head movements [50, 51], or on kinematics and muscle activation patterns during stance and gait behavior [52, 53]. These experiments demonstrated that susceptible individuals standing on the balcony exhibit fewer and amplitude-limited eye-in-head saccades with longer fixation durations. Corresponding reductions in angular head movements were observed in all dimensions, i.e., yaw, pitch, and roll plane (Fig. 2, upper panel). Total gaze-in-space behavior was considerably restricted to a smaller area as compared to non-susceptible controls who freely explored the entire visual field including the abyss [50]. Thus, visual exploration in susceptible individuals confines itself along the horizon or towards a centered position at the horizon. In other words, fear of heights appears to freeze the gaze to the horizon. Finally, experiments revealed an activity-dependent anisotropy of visual exploration in susceptible individuals. In contrast to quiet stance where gaze primarily stays in the horizontal dimension, gaze-in-space behavior during locomotion is mainly restricted to the vertical dimension. This exploration pattern is focused on the ‘vertical strip’ in the heading direction and may augment visual control of balance and avoidance of obstacles in precarious locomotion contexts [51].

With respect to static postural control in susceptible individuals, the relationship between alterations in body sway and muscle activity measures with subjective estimates of fear at heights was examined. Body sway alterations as well as leg- and neck-muscle co-contraction increased with increasing subjective anxiety [52] (Fig. 2, left lower panel). In accordance with the above-described laboratory-based findings in healthy controls, this observation was explained as a fear-induced lowering of the threshold for sensorimotor balance reflexes accompanied by an increased stiffening of the complete postural control apparatus. Analogously, gait alterations of susceptible individuals during real height exposure resemble those seen in healthy subjects when walking on modestly elevated support surfaces, in particular a reduction of walking speed, stride length, and increased double support phases. These are typical features of cautious gait control, a strategy which, however, becomes overridden during walking at fast speeds [53] (Fig. 2, right lower panel).

Taken together, experimental evidence on behavioral changes of gaze, balance, and locomotion control in susceptible individuals during exposure to real heights reveals a stiffening strategy that encompasses the whole anti-gravity musculoskeletal apparatus, including the ocular-motor apparatus. This threat-induced stiffening is associated with a sensitization of sensorimotor balance reflexes, a cautious mode of voluntary motion, and an activity-dependent restriction of visual exploration [54]. These alterations can best be described by the common expression of being ‘scared stiff’ by fear of heights—a behavioral response that results in tonic immobility. This motor response of a tonic stiffening of eye, head, and body movements may represent an atavistic motor reaction resembling feigning death, a primitive reflex observed throughout the entire animal world [54]. Feigning death was first described in snakes by Kilpatrick in 1893 [55]. Accordingly, anxiety appears to be the critical psychopathological symptom that causes the typical, but not specific eye and body motor responses to height exposure in subjects with visual height intolerance.

## Irrational anxiety of falling rather than perception of height is causative

Behavioral responses at heights could be either triggered by anxiety and/or the visual perception of depth. Tersteeg and colleagues [44] directly addressed the question on differential contributions to postural alterations at heights in an experiment on healthy subjects who were walking on a 3.5 m high narrow walkway, while the sight of the drop could be temporarily replaced by a visual surround comparable to ground level. Interestingly, they observed that the mere postural threat by the knowledge of danger rather than the actual perception of height was responsible for the switch to a cautious gait pattern. This is in agreement with the observation that the sensitization of vestibulospinal reflexes at height exposure appears to be primarily driven by anxiety and fear of falling rather than the mere visual perception of depth [46, 47]. Furthermore, changes in balance control and locomotion in individuals susceptible to visual height intolerance were shown to scale with the severity of subjectively perceived anxiety [52, 53]. In contrast, withdrawal of visual depth perception by eye closure or upward gaze only mildly modulates behavioral alterations at heights in these individuals. A recent study in a comprehensive cohort of individuals with different degrees of susceptibility (insusceptible or susceptible up to acrophobic) further suggests that the intensity of perceived anxiety at heights and the extent of corresponding behavioral alterations is directly proportional (Wuehr et al. 2019). Accordingly, the magnitude of postural responses scaled with both, the subjectively felt anxiety during exposure and the severity of individual susceptibility for height intolerance.

**Fig. 3:**
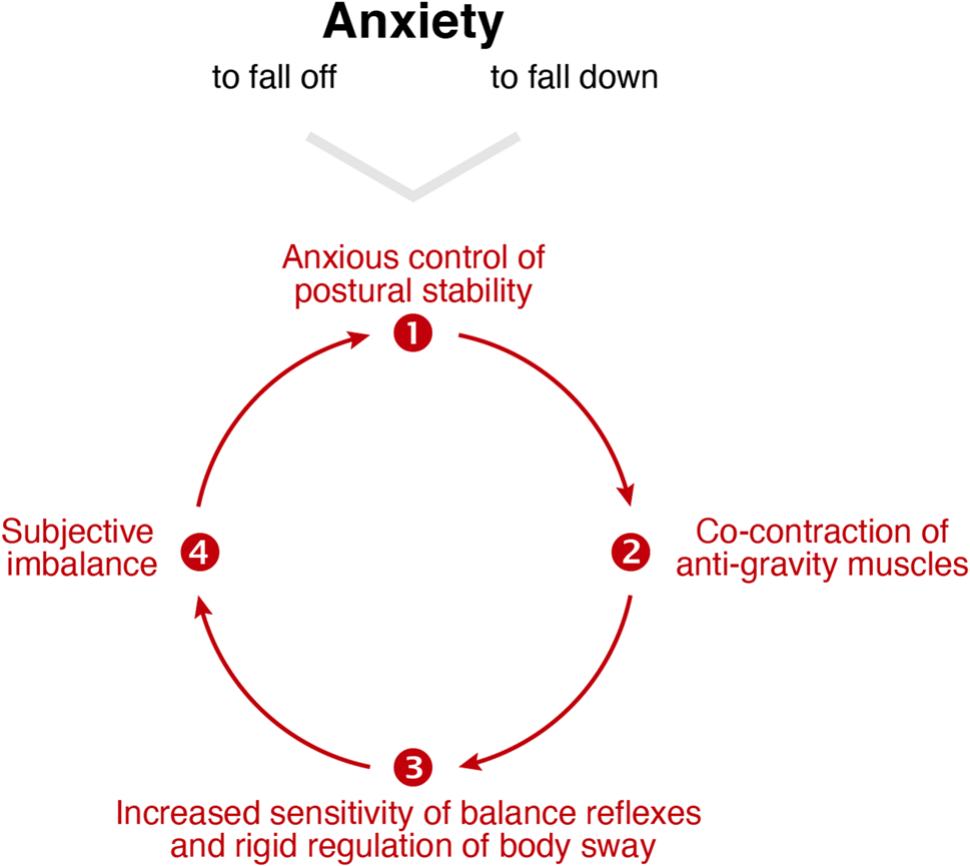
Symptom cascade in persons susceptible to acrophobia and visual height intolerance, the vicious circle or circulus vitiosus. Anxious concentration on control of postural stability triggers co-contraction of the anti-gravity muscles, thereby causing an increased sensitivity of sensorimotor balance reflexes and a rigid regulation of body sway. This leads to subjective imbalance, which in turn enhances anxious control of posture (modified from [54])

Based on this evidence, a hypothetical cascade on emergence of symptoms during height exposure has been proposed [54]. Accordingly, (1) anxiety of falling off or falling down at heights triggers a vicious circle with a (2) co-contraction of anti-gravity muscles that results in (3) increased sensitivity of sensorimotor balance reflexes and a rigid regulation of body sway, which (4) aggravates subjective imbalance and in turn intensifies the initial anxiety [54] (Fig. 3). In line with this proposed symptom cascade, postural changes during height exposure are linked to an increased self-awareness of body sway [56]. There is growing evidence that the vestibular system—by means of reciprocal interconnections within a multilocal anxiety system—influences both cognition and emotional regulation in animal models and humans [57]. This raises the question, whether preserved vestibular function is relevant for distressing anxiety and whether loss of vestibular function reduces general liability to anxious behavior [58]. Indeed, patients with bilateral vestibulopathy—as distinct from other vestibular disorders—have no increased susceptibility to visual height intolerance compared to the general population [26]. In line with this finding, patients with bilateral vestibulopathy do not report anxiety about falling despite having an increased risk of falling [59, 60]. These findings strongly support the impact of anxiety as a trigger and pathophysiological factor of visual height intolerance and acrophobia. However, the severity of anxiety does not always parallel the severity of the neurophysiological parameters of stance and gait as found by Wuehr et al. [61] in a study on the influence of the absolute height above ground on various bodily parameters.

## The role of absolute height above ground on the magnitude of visual height intolerance and acrophobia

**Fig. 4:**
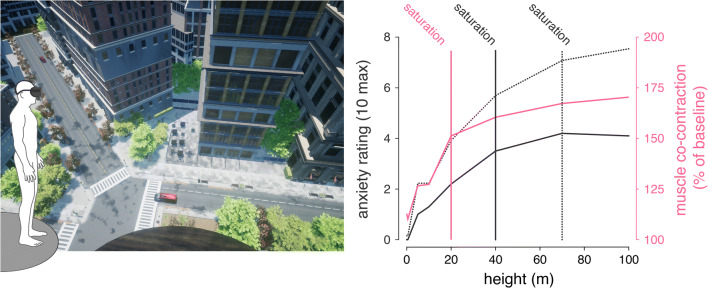
The dependency of anxiety and postural responses on absolute height above ground as studied using virtual reality technology. Left panel: exemplary view of the virtual scene at 40 m above ground. Subjects were exposed to virtual heights ranging from 0 to 100 m via a head-mounted display. Right panel: anxiety ratings (gray solid line: non-acrophobic individuals; gray dotted line: acrophobic individuals) initially increase with increasing height above ground but saturate for heights above 40 m in non-acrophobic and heights above 70 m in acrophobic individuals. In contrast, bodily responses, e.g. muscle co-contraction (pink solid line) saturates already for heights above 20 m in both acrophobic and non-acrophobic individuals

Only a few studies are available on the influence of the increasing heights above ground on the severity of signs and symptoms of visual height intolerance. In psychophysical magnitude estimations, the influence of the net altitude was determined under real conditions on a high building under construction [3]. For most subjects, fear of height was already reported to be highest on the fourth floor and seemingly saturated at a height of about 20 m. Accordingly, there were no significant differences among the intensities of subjective fear of height experienced at altitudes of 20, 50, or 100 m, although some individuals reported increased fear on the 20th floor. This initial observation was later reexamined in more detail by means of virtual reality technology in a comprehensive cohort of individuals with different degrees of susceptibility (insusceptible or susceptible up to acrophobic as differentiated by the Visual Height Intolerance Severity Scale (vHISS [13]) [61] (Fig. 4). Virtual height exposure at seven discrete elevation levels between 0.5 and 100 m provoked similar changes in postural and autonomic measures as observed during real in vivo [61], similar as demonstrated in previous reports [62]. Body sway and musculoskeletal stiffening linearly increased with increasing height up to 20 m above ground beyond which the intensity of postural responses saturated. In contrast, anxiety was found to further increase with height and only saturated above 40 m in non-acrophobic and 70 m in acrophobic participants (Fig. 4). This difference in height-dependency suggests a dissociation between sensorimotor and emotional reactions when being confronted with heights.

## Behavioral recommendations for prevention and therapy of visual height intolerance

Visual height intolerance and acrophobia are characterized by a dissociation between subjective fear and objective danger of falling. Although the thus affected individuals are able to recognize this discrepancy, they can typically overcome inappropriate avoidance behavior only with difficulty. The above-discussed experiments allow recommendation guidelines for coping strategies to avoid or minimize visual height intolerance and acrophobia under natural stimulus height conditions (Table 2). The recommendations for visual and positional behavior are based on the stimulus characteristics required for optimal visual and somatosensory contribution to postural balance [3]. The efficacy of cognitive dual tasking has been convincingly revealed in susceptible individuals during height exposure [52, 53]. Attentional distraction was also shown to improve performance of postural balance in non-susceptible individuals [63]. With respect to psychotherapy, most studies and reviews emphasize that behavioral therapy and its subform cognitive-behavioral therapy are most effective in the treatment of specific phobias such as acrophobia [10]. In particular, exposure therapy approaches for fear of heights are based on the assumption that anxiety and behavioral responses to fear-related stimuli will attenuate within the course of repeated exposures [64]. This treatment approach is in line with therapeutic approaches already used by JW von Goethe in 1771 [65]. In the ninth book of the ‘Straßburger Tischgesellschaft, Sebsterziehung’, he describes how he successfully treated his acrophobia by climbing up daily to the top of the ‘Straßburger Münster’. His technique can be defined as a self-controlled in vivo desensitization between ‘successive approximation’ and ‘flooding’. Whereas there is broad evidence that repeated confrontation with real or virtual fear-inducing height stimuli can results in a rapid remission of anxiety and autonomic responses in afflicted individuals, recent studies indicate that threat-induced changes in balance regulation may largely persist across repeated exposures to heights [61, 66]. Finally, while a wide range of therapies has been proven to be effective in the short term, therapeutic improvements do not persist in the long term in most cases [67].

**Table 2:** Recommendations for behavioral coping strategies for visual height intolerance (modified from [54])

Vision	Fixate the horizon
	Look at near stationary contrasts
	When looking into an abyss, keep near stationary objects in sight in the peripheral field of vision to maintain visual control of posture
	Avoid large-field motion stimuli (for example, clouds) that can lead to visually induced illusory motion
	Do not look through binoculars without some kind of support/stabilization (misleading visual motion stimulus)
	When standing you may close your eyes for a while (to reduce anxiety)
Position	Sit down or lie down (symptoms maximal when standing, minimal when lying)
	Lean on something, hold tight to something
Locomotion	Pause or stop walking (symptoms increase with locomotion at heights)
Cognition	A cognitive dual task (e.g., naming items from a given category) reduces anxiety and improves balance during stance and locomotion
	Try to overcome any avoidance behavior
